# Fear of falling risk after hip surgery in older adults: an updated sex-specific systematic review with GRADE and PAF assessment

**DOI:** 10.1186/s12877-026-07490-y

**Published:** 2026-04-21

**Authors:** Jiachen Ren, Huixian Sun, Ke Wu, Junyu Zhu, Zhanhui Jia, Xiaotong Xu, Huiling Liu

**Affiliations:** The Second Hospital of Hebei Medical University, Shijiazhuang, 050000 Hebei People’s Republic of China

**Keywords:** Hip Fractures, Fear of Falling, Incidence, Risk Factors, Meta-Analysis

## Abstract

**Background:**

Fear of falling (FOF) is a common clinical issue in elderly individuals, especially after hip fractures. Studies show wide variation in FOF prevalence after fractures, with rates ranging from 38 to 72% and notable differences among study groups. Differences in study design and assessment methods make it necessary to conduct a rigorous systematic review. This will consolidate evidence on both epidemiological patterns and modifiable risk factors for FOF.

**Methods:**

A systematic literature search was conducted across nine electronic databases (PubMed, Web of Science, Cochrane Library, Embase, CINAHL, MEDLINE, PsycINFO, CBM, CNKI, VIP, and Wanfang) from inception through January 1, 2025, to identify observational studies investigating the incidence of FOF and associated risk factors in elderly hip fracture patients. Risk of bias assessment was conducted using the Newcastle–Ottawa Scale. Stata 15.0 software was used for meta-analysis, subgroup analysis, and sensitivity analysis in heterogeneity studies. Publication bias was assessed using funnel plots, Egger tests, and Begg tests. We used the population attributable fraction (PAF) and the grading of recommendations, assessment, development, and evaluation (GRADE) evidence assessment to evaluate the quality of the research results.

**Results:**

Nine studies involving 3,088 patients from seven countries were included in the meta-analysis. The results indicated that the incidence of FOF was 66.88% (*95%CI 0.64–0.70, P* < *0.001*) in elderly patients with hip fractures, with a higher incidence observed in developing countries (70.61%) and cases using the simplified International Fall Efficacy Scale (67.00%). The incidence of FOF in female patients (71.95%) was significantly higher than that in male patients (53.97%). Those history of falls (*OR* = *1.98, 95%CI, 1.50–2.24, P* < *0.001*), postoperative pain (*OR* = *1.77, 95%CI, 1.03–3.04, P* = *0.04*), conjunction with other chronic diseases (*OR* = *1.25, 95%CI, 1.00–1.55, P* = *0.05*), poor mobility (*OR* = *1.62, 95%CI, 1.30–2.02, P* = *0.001*), and anxiety (*OR* = *1.13, 95%CI, 1.10–1.15, P* < *0.001*) were identified as risk factors for fear of falling in elderly patients with hip fractures. The results of the evaluation of evidence showed that the history of falls, pain, and anxiety was of high quality. PAF analysis indicated that postoperative pain for 33.99% (*95% CI 4.69–63.97%*) and anxiety for 8.00% (*95% CI 6.54–9.46%*) of FOF.

**Conclusion:**

FOF among older adults remains a substantial global health burden, with notable gendered disparities. If postoperative pain was controlled, approximately one in five FOF could be avoided. Future high-quality longitudinal studies are warranted to refine causal pathways and evaluate the effectiveness of targeted interventions in diverse populations.

**Trial registration:**

This study was registered in the International Database of Prospectively Registered Systematic Reviews (PROSPERO: CRD42025623930).

**Supplementary Information:**

The online version contains supplementary material available at 10.1186/s12877-026-07490-y.

## Introduction

The accelerated global aging of populations has made geriatric hip fractures a major public health challenge [1–3]. Evidence shows one-year post-fracture mortality rates reach 20–30%. Only about 50% of patients recover their pre-fracture function [4]. At the same time, the psychological impacts of fractures strongly influence rehabilitation outcomes. Fear of falling (FOF) is a common complication, with an incidence of 38–72% in this group [5–7]. FOF significantly impairs postoperative recovery and quality of life and raises the risk of future fractures [8].

A history of falls, weak bones, and neuromuscular impairment forms a harmful cycle. Increased caution after a fall may lead to less activity. This decline in activity can worsen function and heighten the risk of falling again [9]. These points highlight why it is critical to identify FOF risk factors early in elderly patients with hip fractures. Yet, present studies show wide differences in both the rates of FOF [5–7] and the profiles of risk factors [10, 11].

This evidence-based investigation aimed to systematically examine FOF incidence patterns and the associated risk determinants in geriatric hip fracture populations. These findings are expected to inform targeted screening protocols and facilitate the development of preventive strategies for high-risk individuals.

## Methods

### Design

This systematic review and meta-analysis were conducted according to the Reporting Items for Systematic Review and Meta-Analysis (PRISMA) guidelines and were registered in the International Database of Prospectively Registered Systematic Reviews [12]. Additionally, the methodological quality of the review process was assessed using the AMSTAR 2 (assessing the methodological quality of systematic reviews) tool [13], a critical appraisal instrument for systematic reviews that include randomized or nonrandomized studies of healthcare interventions.

### Search methods

A computer-based search was conducted using the following databases: PubMed, Web of Science, Cochrane Library, EMBASE, CINAHL, MEDLINE, PsycINFO, CBM, CNKI, VIP, and Wanfang. The search was conducted by combining the subject and free words, and the search time was from the establishment of the database to January 1, 2025. Concurrently, the references included in the study were tracked and screened. The English search term is "older adult", "hip fractures", and "fear of falling". Different database of search strategies was shown in Supplementary Information 1.

### Inclusion and exclusion criteria

After removing duplicate studies, two reviewers independently assessed the remaining studies based on their titles and abstracts. Subsequent literature screening was conducted according to the inclusion and exclusion criteria. The inclusion criteria for the studies were as follows: (i) participants who were at least 60 years old; (ii) the study was included cohort studies; (iii) outcome indicators: the main outcome indicators were various factors affecting fall fear in elderly patients with hip fracture; (iv) the literature provides the adjusted odds ratio (OR) and 95% confidence interval (CI) after multi-factor analysis; OR provides relevant data that can be converted into OR value, 95% CI, and standard error. The exclusion criteria were as follows:(i) reviews, case reports, or meta-analyses, conference minutes, and other studies; (ii) original text that could not be obtained, repeated publication, or incomplete original data.

### Data extraction

Two researchers independently extracted the data using the following variables: first author’s name, publication year, country, type of study instrument, subject, age, sample size, prevalence of FOF, quality of studies, and risk factors for FOF. If the FOF was assessed using different instruments, its prevalence was extracted according to the results of the eligible studies. Disagreements were resolved through discussions between the two researchers, and a third researcher was consulted if required.

### Quality assessment

Assessment of potential bias was conducted by two researchers, and any discrepancies were resolved through discussion or consultation with a third researcher. The quality of cohort studies was assessed using the Newcastle–Ottawa Scale (NOS) [14], a tool with eight items and a total score ranging from 0 to 9. Assessment of potential bias was conducted by two researchers, and any discrepancies were resolved through discussion or consultation with a third researcher.

### Data analysis

A meta-analysis of the included studies was performed using Stata 15.0 software. The OR, 95%CI, and incidence rates in the included studies were pooled to explore the risk factors and incidence of fear of falls in elderly patients with hip fractures. The Chi-square test and *I*^2^ index were used to evaluate heterogeneity. *P* < 0.1 or *I*^2^ > 50% indicates large heterogeneity among studies. A random-effects model was employed for the analysis, and a sensitivity analysis was conducted to ascertain the source of heterogeneity, or a subgroup analysis was conducted where appropriate.

### Evidence evaluation

The GRADE evidence evaluation tool [15] was used to evaluate the included evidence, including the risk of bias, imprecision, inconsistency, incoherence, and five additional domains. The GRADE system categorizes evidence into four levels: high, medium, low, and very low.

### Population attributable fraction analysis

Population attributable fraction (PAF) was estimated for exposure factors rated as high or moderate quality in the GRADE assessment. The standard formula was applied [16]:

$$\mathrm{PAF}=\mathrm{Pe}\times\left(\mathrm{OR}-1\right)/\left[1+\mathrm{Pe}\times\left(\mathrm{OR}-1\right)\right]$$

where Pe represents the overall prevalence of exposure among older adults with FOF following hip fracture surgery in this study, and only exposure factors with high or moderate quality evidence according to the GRADE assessment were included for the OR.

The 95%CIs for the PAF were calculated using the delta method, incorporating the confidence limits of both the OR and Pe. All statistical analyses were performed using the epiR package in R software, version 4.4.2 (R Foundation for Statistical Computing).

## Results

### Study process

**Fig. 1:**
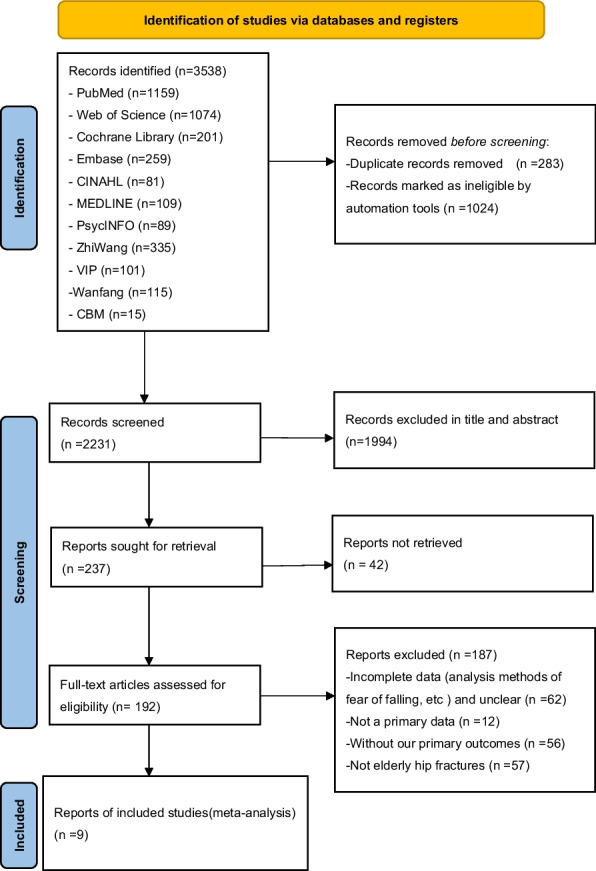
The PRISMA flow diagram showing process of study selection for inclusion in our meta-analyses

Following a thorough screening of titles and abstracts, 237 studies were selected for further analysis. However, subsequent screening of the full text led to the exclusion of 224 studies for the following reasons: inconsistent research object (*n* = 62), inconsistent outcome index (*n* = 68), inconsistent study type (*n* = 88), inability to extract data (*n* = 6), and inability to obtain the full text (*n* = 4). Following the completion of the selection process, 9 studies, encompassing 3,088 participants from seven countries, were included in the analysis (shown in Fig. 1).

### Characteristics of the included studies

Among the 9 articles included in the meta-analysis [7, 17–24], 2 were in Chinese and 7 were in English, with a total of 3,088 patients, the majority of whom were from China, with others from the United States, Finland, Japan, Iran, Turkiye, and Netherlands. The included studies covered the period from 2014 to 2024. The results of the literature quality evaluation showed that 6 articles were of high quality and the rest were of medium quality. Further details are provided in Table 1 and Supplementary Information 6.

**Table 1:** Descriptive characteristics of the included studies

Inclusion study	Research type	Quality evaluation	nation	General population	Evaluation tool	Risk factor
Bower ES, et al. 2016 A [7]	cohort study	7	USA	240	1	①③⑦⑧
Bower ES, et al. 2016 B [7]			USA	152	1	①③⑦
Bower ES, et al. 2016 C [7]			USA	241	1	①③⑦
Bower ES, et al. 2020 [17]	cohort study	9	USA	263	1	⑦⑧
Turhan Damar H, et al. 2018 [18]	cohort study	6	Türkiye	204	2	②④⑤⑨
Jaatinen R, et al. 2022 [19]	cohort study	7	Finland	916	2	①⑤⑥⑦⑧⑩⑫
Nagai K, et al. 2014 [20]	cohort study	6	Japan	214	1	①④⑤⑥⑩
Soleimani R, et al. 2020 [21]	cohort study	8	Iran	88	1	①④
Visschedijk JH, et al. 2014 [22]	cohort study	6	Netherlands	100	1	④⑥⑪⑫
C.Y, et al. 2023 [25]	cohort study	8	China	303	2	②③⑤⑦⑧⑪⑫
W.Y, et al. 2024 [26]	cohort study	8	China	367	1	④⑤⑧

^*^FOF: fear of falling. One is the simplified version of the International Fall Efficacy Scale; 2 is the self-made single choice "there is no fear of falling.” ① is the age; ② for pain; ③ for depression; ④ for anxiety; ⑤ History of falls; ⑥ is HISS score; ⑦ for other chronic diseases; ⑧ for social support; ⑨ is the level of education; ⑩ for mobility and ⑫Activities of daily living (ADL). Because the cohort study by Bower ES et al. reported three groups that met our inclusion criteria, we extracted each group separately and designated them as Bower ES et al. 2016 A, Bower ES et al. 2016 B, and Bower ES et al. 2016 C

### Prevalence of FOF in elderly patients with hip fracture

**Fig. 2:**
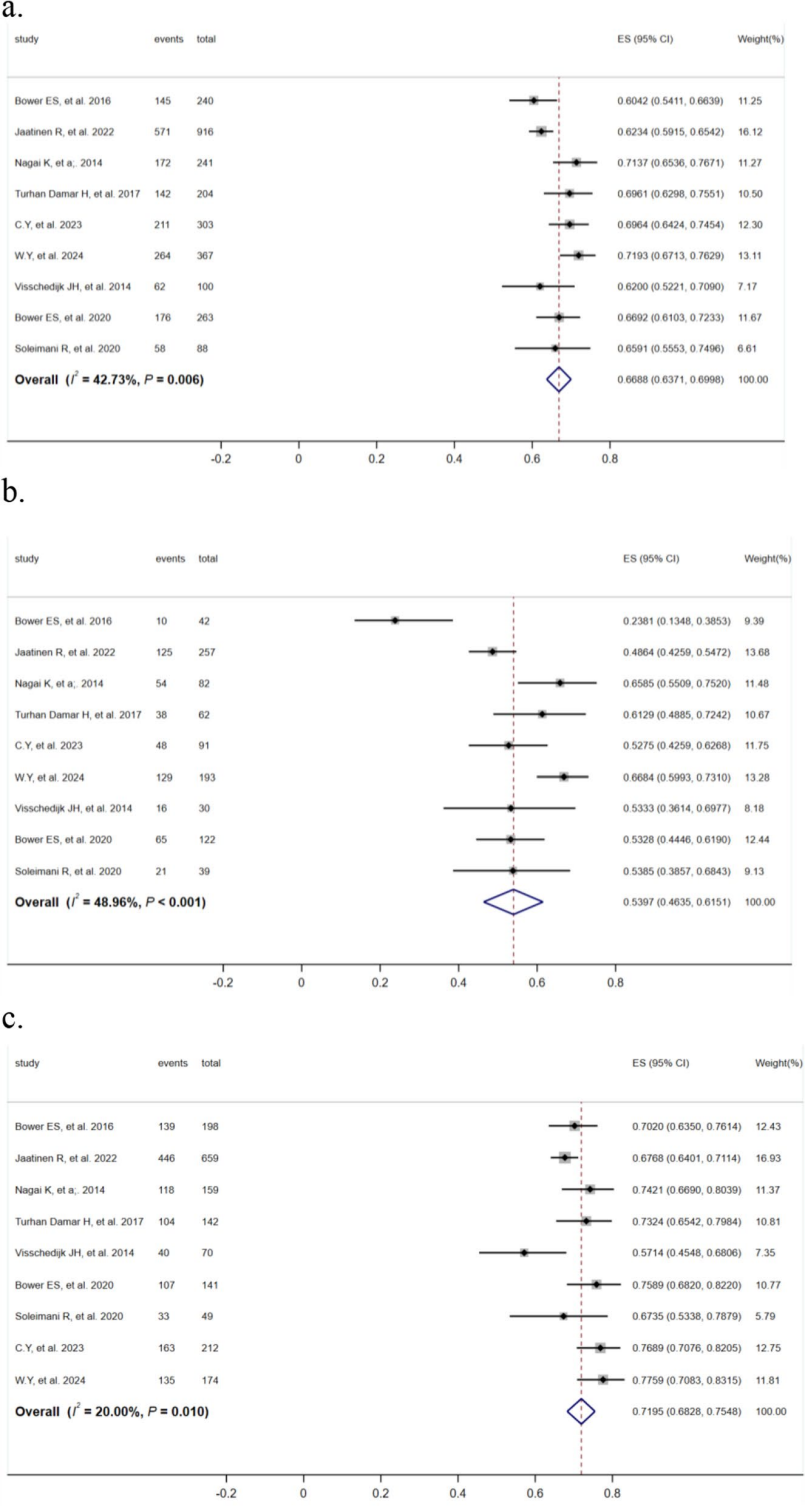
Meta-analysis of the incidence of fear of falling in elderly hip fractures. **a** the general hip fractures; **b** the male hip fractures; **c** the female hip fractures

A meta-analysis was performed on the incidence rates of the 9 included cohort studies [7, 17–24], and significant heterogeneity among the studies was identified (*I*^2^ = *42.73%*, *P* = *0.006*). The fixed-effects model was employed to consolidate the findings, and the results indicated that the prevalence of FOF in elderly patients with hip fracture was 66.88% (*95%CI 0.64–0.70, P* < *0.001*), male was 53.97% (*95%CI 0.46–0.62, P* < *0.001*) and female was 71.95% (*95%CI 0.68–0.75, P* < *0.001*) (Fig. 2). The results of the subgroup analysis showed that the incidence of FOF was higher in elderly hip fracture patients in developing countries and using the simplified International Fall Efficacy Scale as the survey tool (Table 2).

**Table 2:** Subgroup analysis of the incidence of fear of falling in older adults with hip fracture

Item		Number	Heterogeneity test		Effect model	Meta-analysis	
			*I*^*2*^	*P*		Prevalence rate	*95%CI*
National development level	Developed country	6	43.41%	< 0.001	fixed	64.75%	0.61 ~ 0.68
	Developing country	3				70.61%	0.68 ~ 0.74
Evaluation tool	FES-I	6	32.73%	< 0.001	fixed	67.00%	0.63 ~ 0.71
	self-restraint	3				66.74%	0.61 ~ 0.72

### Risk factors for FOF

A meta-analysis was performed on independent variables from more than two relevant studies, encompassing 11 independent variables in the analysis. The results of the meta-analysis showed that fall history, pain, combined with other chronic diseases, poor mobility, and anxiety were risk factors of FOF in elderly patients with hip fractures (Table 3).

**Table 3:** Meta-analysis results and bias test results of risk factors for fear of falling in elderly patients after hip fracture surgery

Factors		Included literature	Sample size	Heterogeneity test		Effect model	Meta-analysis		
				*I*^*2*^	*P*		*OR*	*95%CI*	*P*
Demographic factor	Age	5	2208	94%	< 0.001	Random	1.00	0.93 ~ 1.07	0.94
	Fall history	6	2245	45%	0.020	fixed	1.98	1.50 ~ 2.24	< 0.001
Somatic factor	Pain	3	748	83%	0.003	Random	1.77	1.03 ~ 3.04	0.04
	Combined with other chronic diseases	6	2115	90%	< 0.001	Random	1.25	1.00 ~ 1.55	0.05
	Mobility	3	1130	41%	0.13	fixed	1.62	1.30 ~ 2.02	0.001
	ADL	3	1319	79%	0.009	Random	0.99	0.87 ~ 1.13	0.920
	HISS	3	1230	98%	< 0.001	Random	1.11	0.78 ~ 1.57	0.560
Psychosocial factors	Depressed	5	1303	90%	< 0.001	Random	1.18	0.94 ~ 1.48	0.15
	Anxiety	6	1389	43%	< 0.001	fixed	1.13	1.10 ~ 1.15	< 0.001
	Society support	5	2089	89%	< 0.001	Random	1.05	0.83 ~ 1.32	0.700

*HISS* Hip Injury Severity Score

### Publication bias and sensitivity analysis

The results of the sensitivity analysis showed that the outcomes of the meta-analysis were consistent and reliable (Supplementary Information 2). The funnel plot of the meta-analysis results concerning the incidence of FOF in elderly patients with hip fractures suggests the presence of no-potential publication bias (Supplementary Information 3). Furthermore, the bias tests conducted for each subgroup did not reveal any evidence of a publication bias (Supplementary Information 4). The bias test of various risk factors for FOF in elderly patients with hip fractures suggested no publication bias, as shown in Supplementary Information 5.

### Evidence evaluation

The GRADE evidence evaluation of fall fear risk factors in elderly patients with hip fractures is shown in Table 4. Three risk factors were moderate quality, three were low quality, and four were very low quality. The reduction in quality can be attributed to several factors, and the risk of bias was reduced owing to the presence of bias in the randomized and blind methods of observational studies. The degradation of imprecision was primarily caused by the wide confidence interval resulting from the relatively small number of patients included. The degradation of inconsistency was attributable to the inconsistency of different research results, for which no reasonable explanation could be found. Other significant reasons for the downgrades included potential reporting bias, because the number of included studies was less than five.

**Table 4:** GRADE evaluation and recommendation strength

Risk factors	Included datasets (number)	Quality evidence evaluation					Evidence grade
		Bias risk	Inconsistency	Indirectness	Inexactness	Publishing bias	
Age	5	Yes^a^	Yes^b^	None	Yes^c^	Yes^d^	VERY LOW
Fall history	6	Yes^a^	None	None	Yes^c^	None	MODERATE
Pain	3	Yes^a^	Yes^b^	None	None	None	MODERATE
Combined with other chronic diseases	6	Yes^a^	Yes^b^	None	Yes^c^	Yes^d^	VERY LOW
Mobility	3	Yes^a^	Yes^b^	None	None	Yes^d^	LOW
ADL	3	Yes^a^	Yes^b^	None	Yes^c^	None	LOW
HISS	3	Yes^a^	Yes^b^	None	Yes^c^	Yes^d^	VERY LOW
Depressed	5	Yes^a^	Yes^b^	None	Yes^c^	Yes^d^	VERY LOW
Anxiety	6	Yes^a^	None	None	None	Yes^d^	MODERATE
Society support	5	Yes^a^	Yes^b^	None	Yes^c^	None	LOW

*HISS* Hip Injury Severity Score

^a^Random sequences, allocation concealment, and blinding implementation carry bias risks

^b^There is considerable heterogeneity

^c^The 95% confidence interval is beyond the line

^d^The funnel plot indicates that publication bias may exist

### Population attributable fraction analysis

According to PAF, we found that postoperative pain for 33.99% (*95% CI 4.69–63.97%*) and anxiety for 8.00% (*95% CI 6.54–9.46%*) of FOF. This showed that managing pain after surgery could prevent about one-third of new cases of FOF. Controlling anxiety after surgery may prevent around 8% of these cases.

## Discussion

This study provides novel evidence that the incidence of FOF in elderly patients with hip fractures reaches 66.88%, significantly exceeding the 49.60% observed in the general elderly population [25]. This disparity may stem from the combined effect of post-fracture physical dysfunction and psychological distress. Notably, the higher FOF prevalence in developing countries underscores systemic gaps, including limited access to community rehabilitation services and inadequate provision of mobility aids, which prematurely expose older adults to high-risk activities [27].

The risk of FOF in female patients (71.95%) was significantly higher than that in male patients (53.97%), consistent with the conclusions of Chen et al. [28]. However, the effect size of sex difference in this study was larger, which may be related to slow muscle strength recovery, bone mineral density loss, and social role gap in female patients after hip fracture. Second, this study also found a strong association between fall history and FOF, which was similar to the results reported by Xiong et al. [25], suggesting that the fracture event itself as a traumatic fall may amplify the psychological shadow. Neurobiological studies have further shown that repeated fall experiences can activate the amygdallo-prefrontal cortex fear memory circuit, leading to hypervigilance in daily activities [26]. Third, the influence of multiple chronic diseases on FOF may be realized through multiple mechanisms, which is consistent with the cumulative effect of comorbidities proposed by Quinones et al. [29].

Pain has been identified as the primary physical risk factor for FOF in elderly patients with hip fractures, with its intensity being greater than that reported in ordinary elderly patients with chronic diseases [25]. This suggests that the combination of fracture-related acute and chronic pain may increase the risk of FOF. This finding is consistent with several studies, such as the prospective study of patients with hip fractures by Kalem et al., which showed that the incidence of FOF was significantly higher in patients with persistent postoperative pain than in the pain-free group [30]. The mechanism by which pain drives FOF may involve a vicious cycle of pain-fear-activity avoidance, which has been shown to enhance the perception of fall-related threats by activating nociceptive and emotional integration networks in the anterior cingulate cortex and insula [31] and has been found to lead to compensatory changes in movement patterns (such as increased gait asymmetry), further reducing balance confidence [30].

This study revealed that anxiety is an independent psychosocial risk factor of FOF in elderly patients with hip fractures. First, anxiety has an obvious driving effect on FOF. Consistent with the conclusions of previous studies [25], it is suggested that acute traumatic events after hip fracture may amplify the negative effects of anxiety through the dual stress model: On the one hand, the sudden decline of physical function caused by fracture may lead to heightened vigilance regarding falls. However, postoperative rehabilitation uncertainty may activate the prefrontal amygdala circuit. This exacerbates disaster-based thinking and risk perception [31]. However, the present study did not demonstrate a significant association between depressive symptoms and social support, which is inconsistent with the findings of analogous studies [32]. This difference may be due to the following reasons: (1) Time-dependent effects: depressive symptoms are often masked by somatization complaints (such as pain and fatigue) during the acute phase of hip fracture (≤ 3 months), while FOF assessments are mostly focused on the early postoperative period, which may underestimate the specific effects of depression; (2) Nonlinear threshold of social support: in this study, the social support scale score was right-skewed and probably close to the upper limit of the normal range, suggesting that most patients received high support, whereas only extremely low support levels (such as living alone and without caregivers) significantly affected the risk of FOF.

First, based on high-quality evidence (fall history, pain, and poor walking ability), a three-level risk-warning model was proposed. This model comprises three levels: primary screening (rapid identification of high-risk groups using the FES-I scale upon admission), secondary stratification (risk classification based on chronic disease, anxiety status, and sex differences), and tertiary intervention (individualized rehabilitation programs for mobility function and pain control). Secondly, a time-stratified intervention framework is proposed, with a focus on the management of anxiety in the acute phase and the integration of depression screening with enhanced social support strategies in the middle and later stages of recovery. The core analgesia-function linkage intervention should be established within 0–2 weeks after surgery, with the focus on pain control and anxiety relief. Regional nerve blocks and non-steroidal anti-inflammatory drugs should also be administered. The combination of regional nerve block and non-steroidal anti-inflammatory drugs should be considered to achieve multi-mode analgesia, and progressive out-of-bed training, such as walker-assisted standing, should be implemented to address the chain of bedrest-muscular atrophy-fall risk. Early health education for patients should be emphasized to alleviate their anxiety. After two weeks, the focus should shift to social support and comorbid management.

This study found that the incidence of FOF is higher in elderly patients with hip fractures. Fall history, pain, chronic disease, poor mobility, and anxiety were identified as risk factors for FOF in elderly patients with hip fractures. The factors of fall history, pain, and poor walking should be given particular attention. This study had some limitations. First, the significant heterogeneity among different studies may be due to differences in FOF measurement tools, and future research should focus on establishing standardized FOF measurement tools. Second, in the main study, potential confounding variables, such as socioeconomic status and rehabilitation adherence, were adjusted inconsistently, which may have biased the pooled estimates. Moreover, Calculation of the population attributable fraction (PAF) presupposes a causal relation between the risk factor and fear of falling [33]. Although the restriction to cohort studies strengthens the temporal inference of the observed associations and the results achieved statistical significance, the inherent limitations of observational designs preclude the establishment of causality. Thus, the reported PAF should be interpreted cautiously as the proportion of disease burden that, under the best available evidence, could theoretically be attributed to the exposure, providing a quantitative reference for prioritizing public-health interventions rather than definitive proof of causality. Finally, it is suggested that future studies adopt a variety of machine learning algorithms to build and verify the risk prediction model of FOF in elderly patients with hip fractures and select the optimal model to predict the risk of FOF in elderly patients with hip fractures.

## Conclusion

In summary, FOF among older adults remain a substantial global health burden, with notable gendered disparities. If postoperative pain was controlled, approximately one in five FOF could be avoided. Future high-quality longitudinal studies are warranted to refine causal pathways and evaluate the effectiveness of targeted interventions in diverse populations.

## Supplementary Information

Supplementary Material 1.

## Data Availability

All data from this study can be requested from the corresponding author with a valid reason.
