# Supplementary material for: Fear of falling risk after hip surgery in older adults: an updated sex-specific systematic review with GRADE and PAF assessment
Source: BMC Geriatr. 2026 Apr 21;26:784. doi: 10.1186/s12877-026-07490-y (PMC13227633; doi:10.1186/s12877-026-07490-y)
Supplement: Supplementary file 1 — Supplementary Material 1. [file 12877_2026_7490_MOESM1_ESM.docx]

Supplementary Information 1. Different database of search strategies.

Supplementary Information 2. Sensitivity analysis results graph of overall incidence rate and incidence rate by gender group.

Supplementary Information 3. Funnel plot of overall incidence rate and incidence rate by gender group.

Supplementary Information 4. Bias test for subgroup analysis of the incidence of FOF in elderly patients with hip fractures after surgery.

Supplementary Information 5. The results of the bias test for the risk factors of FOF in elderly patients after hip fracture surgery.

Supplementary Information 6. The results of the quality evaluation of the meta analysis.

Supplementary Information 1. Different database of search strategies.

PubMed

| searching steps | Search | Results |
| --- | --- | --- |
| #1 | ((((Aged [MeSH Terms]) OR (older [Title/Abstract])) OR (older adult [Title/Abstract])) OR (elder [Title/Abstract])) OR (elderly [Title/Abstract])) OR (the aged [Title/Abstract])) | 38,640 |
| #2 | ((fear of falling [Title/Abstract]) OR (falls efficacy [Title/Abstract])) OR (Fear Of Falling [Title/Abstract])) OR (concern about falling [MeSH Terms])) | 3,440 |
| #3 | ((hip fractures [MeSH Terms]) OR (trochanteric fractures [Title/Abstract])) OR (hip, fracture [Title/Abstract])) OR (intertrochanteric fractures [Title/Abstract])) OR (subtrochanteric fractures [Title/Abstract])) OR (femoral neck fracture [Title/Abstract])) OR (femoral intertrochanteric fracture [Title/Abstract])) | 66,708 |
| #4 | ((influence factors [MeSH Terms]) OR (risk factors [Title/Abstract])) OR ((relative factors [Title/Abstract]) OR (related factors [Title/Abstract]) OR (relevant factors [Title/Abstract])) | 336,708 |
| #5 | #1 AND #2 AND #3 AND #4 | 1,159 |

Web of Science

| searching steps | Search | Results |
| --- | --- | --- |
| #1 | "(((TS=(Aged)) OR TS=(older)) OR TS=(older adult)) OR TS=(elder)" | 84,199 |
| #2 | "((TS=(fear of falling)) OR TS=(Fear Of Falling)) OR TS=(concern about falling)" | 3,690 |
| #3 | "(((((TS=(hip fractures)) OR TS=(trochanteric fractures)) OR TS=(intertrochanteric fractures )) OR TS=(subtrochanteric fractures)) OR TS=(femoral neck fracture)) OR TS=(femoral intertrochanteric fracture)" | 30,844 |
| #4 | "(((((TS=(influence factors)) OR TS=(risk factors)) OR TS=(relative factors)) OR TS=(related factors)) OR TS=(relevant factors)) " | 471,431 |
| #5 | #1 AND #2 AND #3 AND #4 | 1,074 |

Embase

| searching steps | Search | Results |
| --- | --- | --- |
| #1 | ' Aged'/exp OR ' older':ab,ti OR ' older adult':ab,ti OR ' elder' OR ' elderly':ab,ti | 7,811 |
| #2 | ' fear of falling':ab,ti OR ' falls efficacy':ab,ti OR ' Fear Of Falling':ab,ti OR ' concern about falling':ab,ti | 1,914 |
| #3 | 'hip fractures '/exp OR 'trochanteric fractures ':ab,ti OR 'hip fractures ':ab,ti OR 'intertrochanteric fractures ':ab,ti OR 'subtrochanteric fractures ':ab,ti OR 'femoral neck fracture ':ab,ti OR 'femoral intertrochanteric fracture ':ab,ti | 1,153 |
| #4 | ' influence factors':ab,ti OR ' risk factors':ab,ti OR ' relative factors':ab,ti OR ' related factors':ab,ti OR ' relevant factors ':ab,ti | 8,752 |
| #5 | #1 AND #2 AND #3 AND #4 | 259 |

PsycINFO

| searching steps | Search | Results |
| --- | --- | --- |
| #1 | MA Aged OR AB Aged OR AB older OR AB older adult OR AB elder OR AB elderly | 4,526 |
| #2 | AB fear of falling OR AB falls efficacy OR AB Fear Of Falling OR AB concern about falling | 548 |
| #3 | MA hip fractures OR AB hip fractures OR AB trochanteric fractures OR AB intertrochanteric fractures OR AB subtrochanteric fractures OR AB femoral neck fracture OR AB femoral intertrochanteric fracture | 1,295 |
| #4 | AB influence factors OR AB risk factors OR AB relative factors OR AB related factors OR AB relevant factors | 5,125 |
| #5 | #1 AND #2 AND #3 AND #4 | 89 |

Cochrane Library

| searching steps | Search | Results |
| --- | --- | --- |
| #1 | MeSH descriptor: [Aged] explode all trees | 2,748 |
| #2 | ("Aged"):ti,ab,kw OR ("older"):ti,ab,kw OR ("older adult"):ti,ab,kw OR ("elder"):ti,ab,kw | 1,153 |
| #3 | MeSH descriptor: [hip fractures] explode all trees | 691 |
| #4 | ("hip fractures"):ti,ab,kw OR ("trochanteric fractures"):ti,ab,kw OR ("intertrochanteric fractures"):ti,ab,kw OR ("subtrochanteric fractures "):ti,ab,kw OR ("femoral neck fracture "):ti,ab,kw OR ("femoral intertrochanteric fracture"):ti,ab,kw | 2,104 |
| #5 | MeSH descriptor: [influence factors] explode all trees | 743 |
| #6 | ("influence factors"):ti,ab,kw OR ("risk factors"):ti,ab,kw OR ("relative factors"):ti,ab,kw OR ("related factors"):ti,ab,kw OR ("relevant factors "):ti,ab,kw | 1,907 |
| #7 | ("fear of falling "):ti,ab,kw OR ("falls efficacy "):ti,ab,kw OR ("Fear Of Falling"):ti,ab,kw OR ("concern about falling"):ti,ab,kw | 333 |
| #8 | #1 OR #2 | 3,901 |
| #9 | #3 OR #4 | 2,795 |
| #10 | #5 OR #6 | 2,650 |
| #11 | #7 AND #8 AND #9 AND #10 | 201 |

CINAHL

| searching steps | Search | Results |
| --- | --- | --- |
| #1 | Aged [MH] OR Aged [TT] OR older [TT] OR older adult [TT] OR elder [TT] OR elderly [TT] | 4,526 |
| #2 | fear of falling [TT] OR falls efficacy [TT] OR Fear Of Falling [TT] OR concern about falling[TT] | 119 |
| #3 | hip fractures [MH]OR hip fractures [TT] OR trochanteric fractures [TT] OR intertrochanteric fractures [TT] OR subtrochanteric fractures [TT] OR femoral neck fracture [TT] OR femoral intertrochanteric fracture [TT] | 295 |
| #4 | influence factors [TT] OR risk factors [TT] OR relative factors [TT] OR related factors [TT] OR relevant factors [TT] | 1,025 |
| #5 | #1 AND #2 AND #3 AND #4 | 81 |

MEDLINE

| searching steps | Search | Results |
| --- | --- | --- |
| #1 | Aged [Mesh] OR Aged [tiab] OR older [tiab] OR older adult [tiab] OR elder [tiab] OR elderly [tiab] | 4,666 |
| #2 | fear of falling [tiab] OR falls efficacy [tiab] OR Fear Of Falling [tiab] OR concern about falling[tiab] | 248 |
| #3 | hip fractures [Mesh]OR hip fractures [tiab] OR trochanteric fractures [tiab] OR intertrochanteric fractures [tiab] OR subtrochanteric fractures [tiab] OR femoral neck fracture [tiab] OR femoral intertrochanteric fracture [tiab] | 1,695 |
| #4 | influence factors [tiab] OR risk factors [tiab] OR relative factors [tiab] OR related factors [tiab] OR relevant factors [tiab] | 5,005 |
| #5 | #1 AND #2 AND #3 AND #4 | 109 |

CNKI

| searching steps | Search | Results |
| --- | --- | --- |
| #1 | SU = (' hip fracture '+' hip fractures' + 'fracture between rotor' + 'intertrochanteric fractures' +' femoral neck fractures' + 'femoral fractures between rotor' + 'hip replacement' + 'total hip replacement' + 'hip replacement' + 'hip arthroplasty') | 4,518 |
| #2 | SU = (' Aged '+' older '+' older adult '+' elder '+' elderly '+' the aged') | 3,544 |
| #3 | SU = (' Fall of Fear '+' fear of falling '+' worry about fall '+' fall efficiency) | 515 |
| #4 | SU = (' related factors' + 'influence factors' +' risk factors' + 'risk factors') | 6,157 |
| #5 | #1 AND #2 AND #3 AND #4 | 335 |

Wan Fang

| searching steps | Search | Results |
| --- | --- | --- |
| #1 | Subject =(hip fracture OR hip fracture OR intertrochanteric fracture OR intertrochanteric fracture OR femoral neck fracture OR intertrochanteric fracture OR hip arthroplasty OR total hip arthroplasty OR hip arthroplasty OR total hip arthroplasty OR hip arthroplasty OR total hip arthroplasty) | 2,056 |
| #2 | subject = (Aged OR older OR older adult OR elder OR elderly OR the aged) | 32,045 |
| #3 | subject =(Fear of falling OR Fear of falling OR worry about falling OR fall efficacy) | 335 |
| #4 | subject =(related factors OR influencing factors OR risk factors OR high risk factors) | 3,005 |
| #5 | #1 AND #2 AND #3 AND #4 | 115 |

VIP

| searching steps | Search | Results |
| --- | --- | --- |
| #1 | M=(hip fracture OR hip fracture OR intertrochanteric fracture OR intertrochanteric fracture OR femoral neck fracture OR intertrochanteric fracture OR hip arthroplasty OR total hip arthroplasty OR hip arthroplasty OR total hip arthroplasty) | 3,516 |
| #2 | M= (Aged OR older OR older adult OR elder OR elderly OR the aged) | 4,558 |
| #3 | M=(Fear of falling OR Fear of falling OR worry about falling OR fall efficacy) | 195 |
| #4 | M=(related factors OR influencing factors OR risk factors OR high risk factors) | 5,635 |
| #5 | #1 AND #2 AND #3 AND #4 | 101 |

CBM

| searching steps | Search | Results |
| --- | --- | --- |
| #1 | Subject =(hip fracture OR hip fracture OR intertrochanteric fracture OR intertrochanteric fracture OR femoral neck fracture OR intertrochanteric fracture OR hip arthroplasty OR total hip arthroplasty OR hip arthroplasty OR total hip arthroplasty OR hip arthroplasty OR total hip arthroplasty) | 6,511 |
| #2 | subject = (Aged OR older OR older adult OR elder OR elderly OR the aged) | 39,958 |
| #3 | subject =(Fear of falling OR Fear of falling OR worry about falling OR fall efficacy) | 66 |
| #4 | subject =(related factors OR influencing factors OR risk factors OR high risk factors) | 6,155 |
| #5 | #1 AND #2 AND #3 AND #4 | 15 |

a.


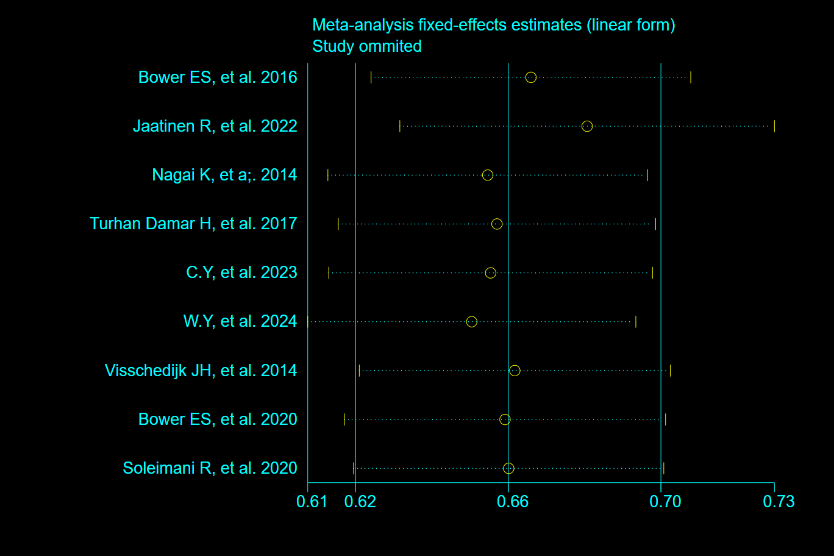


b.


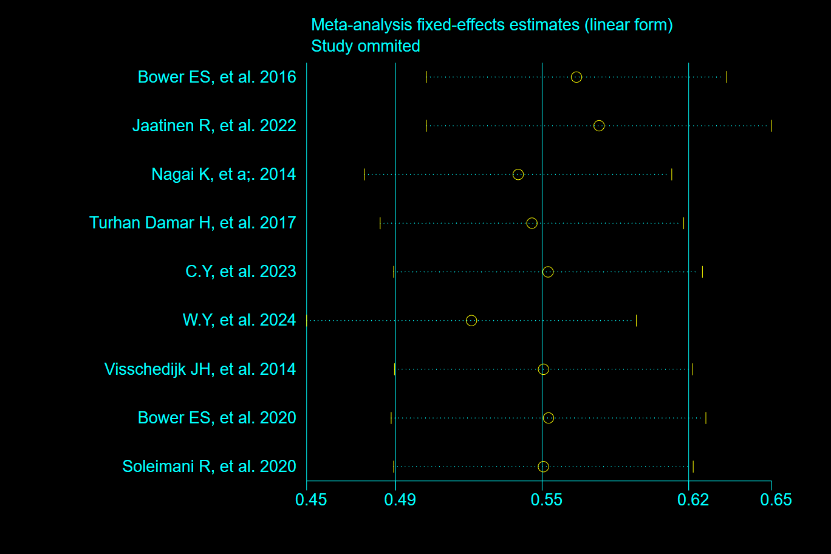


c.


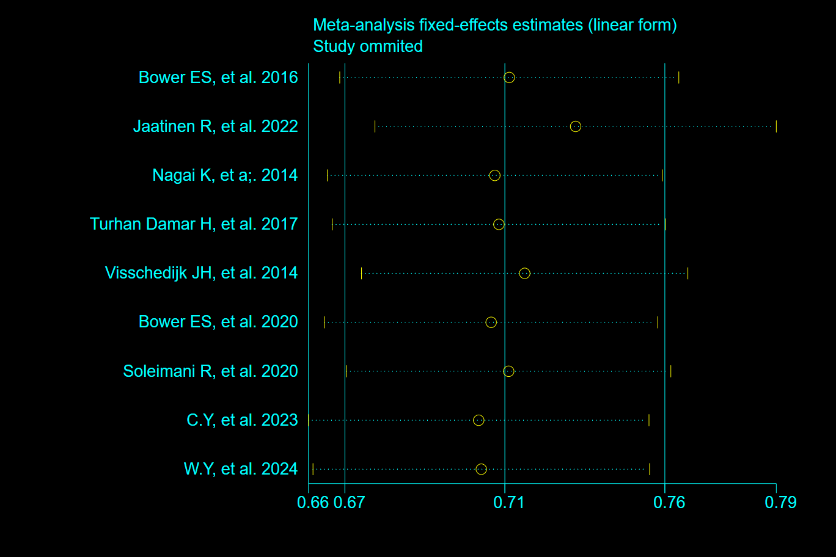


Supplementary Information 2. Sensitivity analysis results graph of overall incidence rate and incidence rate by gender group.

a, overall incidence rate of FOF;

b, male incidence rate of FOF;

c, female incidence rate of FOF.

FOF, Fear of Falling.

a.


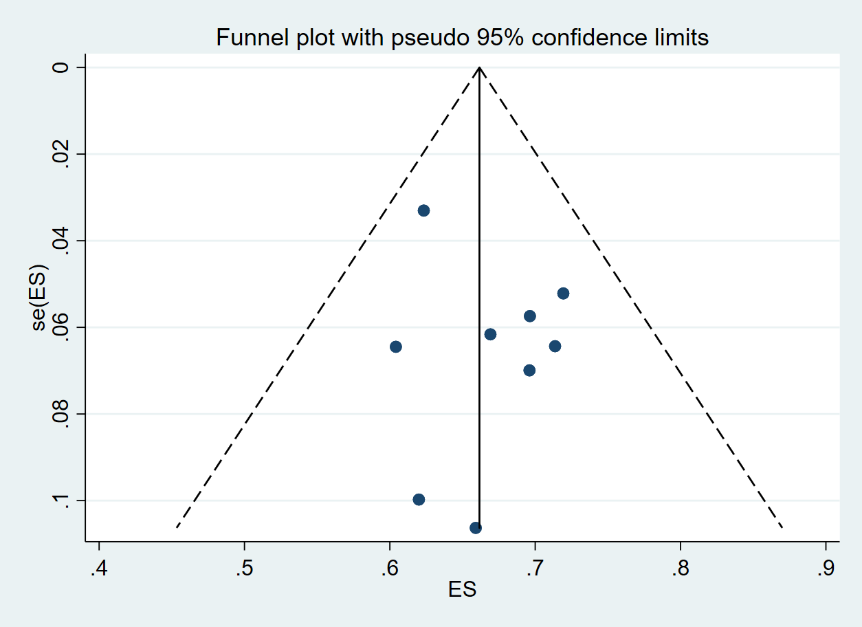


b.


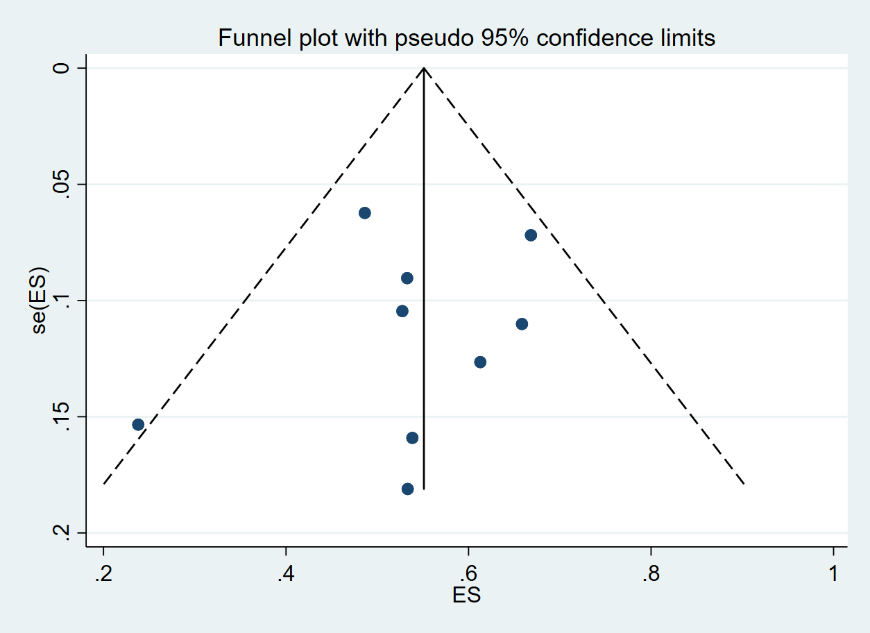


c.


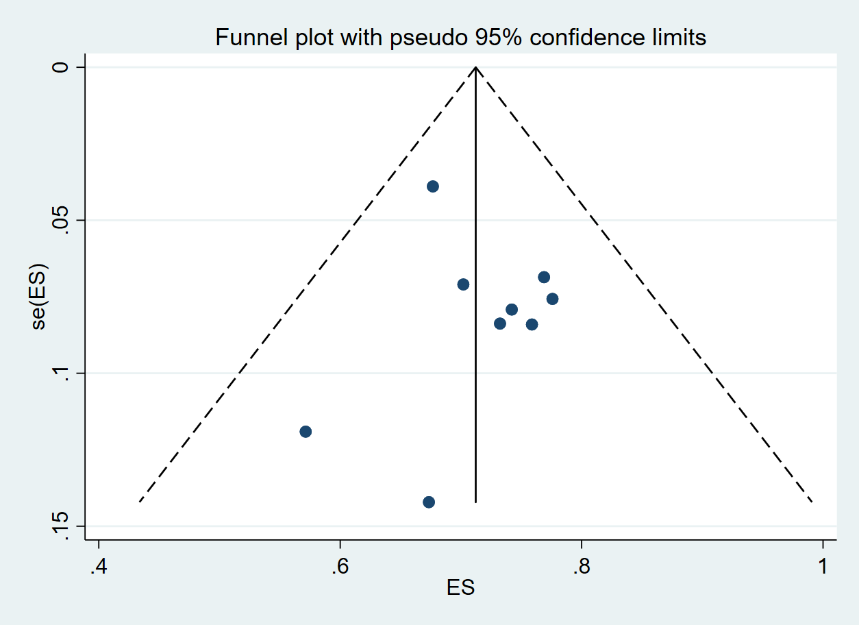


Supplementary Information 3. Funnel plot of overall incidence rate and incidence rate by gender group.

a, overall incidence rate of FOF;

b, male incidence rate of FOF;

c, female incidence rate of FOF.

FOF, Fear of Falling.

Supplementary Information 4. Bias test for subgroup analysis of the incidence of FOF in elderly patients with hip fractures after surgery.

| Item | | Included datasets (number) | Begg's text | Eegger's text |
| --- | --- | --- | --- | --- |
|  |  |  |  |  |
| overall incidence rate of FOF | | 9 | 0.602 | 0.440 |
| male incidence rate of FOF | | 9 | 0.917 | 0.616 |
| female incidence rate of FOF | | 9 | 0.602 | 0.703 |
| National development level | Developed country | 6 | 0.707 | 0.518 |
|  | Developing country | 3 | 0.993 | 0.468 |
| Evaluation tool | FES-I | 6 | 0.260 | 0.333 |
|  | self-restraint | 3 | 0.983 | 0.164 |

FOF, Fear of Falling.

Supplementary Information 5. The results of the bias test for the risk factors of FOF in elderly patients after hip fracture surgery.

| Item | | Included datasets (number) | Begg's text | Eegger's text |
| --- | --- | --- | --- | --- |
|  |  |  |  |  |
| Demographic factor | Age | 5 | 0.515 | 0.338 |
|  | Fall history | 6 | 0.296 | 0.465 |
| Somatic factor | Pain | 3 | 0.771 | 0.561 |
|  | Combined with other chronic diseases | 6 | 0.228 | 0.159 |
|  | Mobility | 3 | 0.096 | 0.091 |
|  | ADL | 3 | 0.988 | 0.736 |
|  | HISS | 3 | 0.977 | 0.634 |
| Psychosocial factors | Depressed | 5 | 0.806 | 0.101 |
|  | Anxiety | 6 | 0.885 | 0.342 |
|  | Society support | 5 | 0.462 | 0.502 |

*FOF, Fear of Falling; HISS, Hip Injury Severity Score.

Supplementary Information 6. The results of the quality evaluation of the meta analysis.

| Inclusion study | Selection | | | | Comparability | Outcome | | | Total |
| --- | --- | --- | --- | --- | --- | --- | --- | --- | --- |
|  | Representativeness of the exposed cohort | Selection of the non exposed cohort | Ascertainment of exposure | Demonstration that outcome of interest was not present at start of study | Comparability of cohorts on the basis of the design or analysis | Assessment of outcome | Was follow-up long enough for outcomes to occur | Adequacy of follow up of cohorts |  |
| Bower ES, et al. 2016 | truly representative of the average | drawn from the same community as the exposed cohort | secure record | yes | study controls for FOF | independent blind assessment | yes | no statement | 7 |
| Bower ES, et al. 2020 | truly representative of the average | drawn from the same community as the exposed cohort | secure record | yes | study controls for FOF and any additional factor | record linkage | yes | complete follow up- all subjects accounted for FOF | 9 |
| Turhan Damar H, et al. 2018 | truly representative of the average | drawn from a different source | secure record | yes | study controls for FOF | independent blind assessment | yes | no statement | 6 |
| Jaatinen R, et al. 2022 | truly representative of the average | drawn from the same community as the exposed cohort | secure record | yes | study controls for FOF | record linkage | yes | no statement | 7 |
| Nagai K, et al. 2014 | truly representative of the average | drawn from the same community as the exposed cohort | secure record | yes | study controls for FOF | self report | yes | no statement | 6 |
| Soleimani R, et al. 2020 | truly representative of the average | drawn from the same community as the exposed cohort | secure record | yes | study controls for FOF and any additional factor | record linkage | yes | no statement | 8 |
| Visschedijk JH, et al. 2014 | truly representative of the average | drawn from a different source | secure record | yes | study controls for FOF | independent blind assessment | yes | no statement | 6 |
| C.Y, et al. 2023 | truly representative of the average | drawn from the same community as the exposed cohort | secure record | yes | study controls for FOF and any additional factor | independent blind assessment | yes | no statement | 8 |
| W.Y, et al. 2024 | truly representative of the average | drawn from the same community as the exposed cohort | secure record | yes | study controls for FOF | record linkage | yes | complete follow up- all subjects accounted for FOF | 8 |
